# Identification of AP2/ERF transcription factors in *Tetrastigma hemsleyanum* revealed the specific roles of ERF46 under cold stress

**DOI:** 10.3389/fpls.2022.936602

**Published:** 2022-08-09

**Authors:** Zhuomi Xie, Chuyun Yang, Siyi Liu, Mingjie Li, Li Gu, Xin Peng, Zhongyi Zhang

**Affiliations:** ^1^College of Agriculture, Fujian Agriculture and Forestry University, Fuzhou, China; ^2^Key Laboratory of Ministry of Education for Genetics, Breeding and Multiple Utilization of Crops, Fujian Agriculture and Forestry University, Fuzhou, China; ^3^Ningbo Municipal Hospital of TCM, Affiliated Hospital of Zhejiang Chinese Medical University, Ningbo, China; ^4^Medicinal Plant Resource Center, Ningbo Research Institute of Traditional Chinese Medicine, Ningbo, China

**Keywords:** *Tetrastigma hemsleyanum Diels at Gilg*, cold stress, expression profiling, AP2/ERF transcription factor (TFs), functional analysis (FA)

## Abstract

*Tetrastigma hemsleyanum* (*T. hemsleyanum*) is a traditional medicinal plant that is widely used in China. Cultivated *T. hemsleyanum* usually encounters cold stress, limiting its growth and quality at key developmental stages. APETALA2 (AP2)/ethylene-responsive factor (ERF) transcription factors (TFs) comprise one of the largest gene superfamilies in plants and are widely involved in biotic and abiotic stresses. To reveal the roles of AP2/ERF TFs during *T. hemsleyanum* development, 70 AP2/ERF TFs were identified in *T. hemsleyanum*. Among them, 18 and 2 TFs were classified into the AP2 and RAV families, respectively. The other 50 TFs belonged to the ERF family and were further divided into the ERF and (dehydration reaction element binding factor) DREB subfamilies. The ERF subfamily contained 46 TFs, while the DREB subfamily contained 4 TFs. Phylogenetic analysis indicated that AP2/ERF TFs could be classified into five groups, in which 10 conserved motifs were confirmed. Several motifs were group- or subgroup-specific, implying that they were significant for the functions of the AP2/ERF TFs of these clades. In addition, 70 AP2/ERF TFs from the five groups were used for an expression pattern analysis under three low-temperature levels, namely, –4, 0, and 4°C. The majority of these AP2/ERF TFs exhibited a positive response to cold stress conditions. Specifically, ThERF5, ThERF31, ThERF46, and ThERF55 demonstrated a more sensitive response to cold stress. Moreover, AP2/ERF TFs exhibited specific expression patterns under cold stress. Transient overexpression and RNA interference indicated that ThERF46 has a specific tolerance to cold stress. These new insights provide the basis for further studies on the roles of AP2/ERF TFs in cold stress tolerance in *T. hemsleyanum*.

## Introduction

*Tetrastigma hemsleyanum* (*T. hemsleyanum*) is one of the traditional medicinal plants that is widely used in China. The root tuber of *T. hemsleyanum* has unique antiviral, antitumor, and anti-inflammatory effects. The growth and development of cultivated *T. hemsleyanum* are mainly affected by abiotic stress, with cold stress being the key environmental factor limiting its growth and quality. Transcription factors (TFs), which can specifically regulate the gene expression of genes by binding to them (Dimova et al., 2003), play critical roles in related regulatory networks or signaling pathways responding to abiotic stress (Sharma et al., 2010).

The APETALA2/ethylene-responsive factor (AP2/ERF) is one of the largest TF families in plants. The protein encoded by the AP2/ERF TF is composed of 60–70 amino acids, mainly including a DNA binding domain, a transcriptional activation or inhibition domain, an oligomerization site, and a nuclear localization signal (Nakano et al., 2006). The biological functions of the AP2/ERF gene family have been extensively studied in model plants. The AP2/ERF family has been shown to play an important role in plant growth, development (Kubo and Kakimoto, 2000; Rashotte et al., 2006; Dietz et al., 2010), and abiotic stress response (Liu et al., 1998; Xu et al., 2011). Moreover, AP2/ERF TFs have been implicated in the plant defense response, signal transmission, stress response, gene expression regulation, plant growth, and developmental regulation. In these processes, different domains of the AP2/ERF family play different roles. The AP2/ERF family is divided into four subfamilies according to the characteristics of their conserved domains, namely, ethylene reaction element binding factor (ERF), dehydration reaction element binding factor (DREB), AP2 related to ABI3/VP1 (RAV), and soloist. Both ERF and DREB subfamily members contain an AP2 domain (Sharma et al., 2010). The Ap2 domain can regulate the binding activity of *cis*-acting elements, such as the dehydration response element/C repeat (DRE/CRT) and GCC box in the promoter region of AP2/ERF TFs’ target genes (Ohme-Takagi and Shinshi, 1995; Jofuku et al., 2005). Ap2 subfamily members contain two adjacent AP2 domains, whereas RAV subfamily members have both an AP2 domain and a B3 domain (Nakano et al., 2006). The whole-genome identification and analysis of the AP2/ERF family have been carried out in several plant species, and AP2/ERF family has been deeply studied in many plants, including *Arabidopsis* (Nakano et al., 2006), rice (Sharoni et al., 2011), alfalfa (Shu et al., 2016), and other plants (Zhuang et al., 2008, 2011; Licausi et al., 2010; Pirrello et al., 2012; Song et al., 2013).

The AP2 subfamily is involved in regulating plant flowering time (Jofuku et al., 1994), flower organ growth and development (Aukerman and Sakai, 2003), ovule development (Klucher et al., 1996), determining spikelet meristem formation (Guillaumot et al., 2008), and leaf epidermal cell formation (Moose and Sisco, 1996). However, the AP2/ERF gene family is regulated by hormones such as ethylene and brassinolide, and its DNA-binding domain can directly interact with the GCC box to regulate the expression of downstream target genes (Lata et al., 2014; Xu et al., 2020). Overexpression of the *AP2/ERF* gene can improve the resistance of plants to salt stress and drought stress (Sohn et al., 2006; Li et al., 2015). AP2/ERF TFs are also involved in salt stress (Wu et al., 2008) and high- and low-temperature stress (Ito et al., 2006; Qin et al., 2007). These findings have greatly promoted the understanding of the biological function of AP2/ERF family TFs. Moreover, numerous studies have identified the roles of AP2/ERF TFs in plants, but the role of AP2/ERF TFs in *T. hemsleyanum* is still unclear.

This study was the first to identify AP2/ERF TFs in *T. hemsleyanum* using a bioinformatics approach. Moreover, it analyzed in detail the expression pattern of the AP2/ERF gene family under cold stress. The functions of the AP2/ERF gene were identified in detail by transient overexpression and RNA-interference expression. The results provide new insights for further investigation of the molecular mechanism of AP2/ERF TFs in *T. hemsleyanum* under cold stress.

## Materials and methods

### Plant materials and stress treatments

Healthy cutting seedlings of *T. hemsleyanum* were grown under standard greenhouse conditions set as follows: a 16-h day/8-h night cycle, a 23 ± 2°C temperature, and 50% relative humidity. Then, 6-month-old seedlings of *T. hemsleyanum* with identical growth potentials were selected for a transient expression experiment of purpose genes. *Agrobacterium tumefaciens* strain GV3101 containing a 35S promoter was used to transiently overexpress or interfere with AP2/ERFs. The density of the GV3101 bacterium solution was amplified to OD600 = 1.0 and set to 5,000/rpm for 5 min. The bacteria were resuspended with the infection solution (containing 100 μM acetosyringone, 10 mM 2-morpholinoethanesulfonic acid, 10 mM MgCl_2_, and pH = 5.7), and the concentration of the solution was adjusted to OD600 = 1.0. The infection solution was injected into the back of leaves by syringe, and a total of 25 plants were infected as repeats. After 72 h of infection, the expression levels of *ThERF46* were detected to determine whether the gene was expressed or silenced by qRT-PCR. When the transient expression was successful, *T. hemsleyanum* was treated at 4°C for 6 h. Phenotypic characteristics and electrolyte leakage were carefully analyzed. Simultaneously, the leaves were sampled, immediately frozen in liquid nitrogen, and stored at –80°C for further analysis on gene expression and biochemical index. The gene expression and biochemical index analysis were repeated three times, and each biological replicate consisted of a sample pool of 25 seedlings.

### Identification of AP2/ERF transcription factors in *Tetrastigma hemsleyanum*

Transcriptome data were obtained in our previous study (accession number: PRJNA797653) (Xie et al., 2022). AP2/ERF TF family proteins of *Arabidopsis thaliana* were compared with the *T. hemsleyanum* transcriptome data, and the *T. hemsleyanum* transcriptome was searched using the HMMER software domain. A hidden Markov model (HMM) of the AP2 (PF00847) was downloaded from the Pfam database^1^ (El-Gebali et al., 2019), and the AP2/ERF family TFs were identified using HMMER3 (version 3.0) software (Eddy, 2011) with a defined threshold of E < 1e^–5^. The NCBI Conserved Domain Search Service (CD Search) (Marchler-Bauer et al., 2017) was used to confirm manually the predicted AP2/ERF family TFs.

### Conserved motif and evolutionary relationship analysis

The conserved motifs in the *T. hemsleyanum* AP2/ERF TFs were identified using the online motif finding tool MEME 4.11.2^2^ (Bailey et al., 2009). *Arabidopsis* and *Vitis vinifera* ERF-related proteins were downloaded from the TAIR website^3^ and NCBI, respectively. The evolutionary relationship of three kinds of ERF-related proteins, namely, AtERF, VvERF, and ThERF, was analyzed using the MEGA 7.0 software.

### Subcellular localization prediction and gene structure analysis

The subcellular localization and physicochemical properties of ThERF proteins were analyzed using the Wolf PSORT software^4^ and ProtParam software^5^, respectively. Three-dimensional models of the ThERF proteins were analyzed using the SWISS-MODEL online software^6^.

### Expression pattern analysis

Total RNA was isolated from 100 mg of leaves (fresh-weight) using a plant RNA extraction kit (Nanjing Vazyme Biotech Co., Ltd.). cDNA synthesis was performed with the Evo M-MLV Mix Kit with gDNA Clean for qPCR AG11728 [Accurate Biotechnology (Hunan) Co., Ltd.]. Each reaction contained 10 μl of 2*SYBR Green Pro Taq HS Premix AG11701 [Accurate Biotechnology (Hunan) Co., Ltd.], 2 μl of template cDNA, and 0.4 μl of each forward and reverse primers (10 μM). The *GAPDH* gene was used as an internal reference. All primers used in this assay are shown in Supplementary Table S1. The PCR reaction procedure was performed as follows: incubation at 95°C for 2 min followed by 40 cycles at 95°C for 5 s and 60°C for 30 s. Each gene was tested in biological triplicates with three technical repeats. The expression level for each sample was expressed as 2^–ΔΔ*CT*^. The data were exhibited as the mean ± SD of three independent experiments.

### Construction of expression vectors and subcellular localization

To construct a transient overexpression vector for *ThERF46*, its full-length of ORF was cloned into the entry vector pBI121-GFP digested by *Kpn*I using the following primers: *ThERF*-forward, 5′ggtaccATGGCGGTCGAGCCCCTC3′ and *ThERF*-reverse, and 5′ggtaccTCAGAAGAGCGGGGCCGG3′. To build an RNA-interference vector for *ThERF46*, a fragment from ORF of *ThERF46* was amplified using the following primers: *ThERF*-forward, 5′accag gtctcaggagATCTCATCCTTCCCGTTCTG3′ and *ThERF*-reverse, and 5′accaggtctcatcgtGGCAGCATCGTCGTAAGC3′. This fragment was then inserted into the entry vector pRNAiGG. To detect the subcellular localization of *ThERF46*, its ORF was further inserted into the pBI121-GFP vector to form fused proteins with GFP. The expression of ThERF46*-*GFP fusion was driven by double 35S promoters. *Agrobacterium tumefaciens* strain GV3101 containing 35S: ThERF46: GFP and 35S: GFP were grown overnight in LB culture solution, and then resuspended to OD600 = 1.0. *Agrobacterium tumefaciens* strains harboring the GFP fusion constructs were filtrated into *N. benthamiana* leaves.

### Biochemical index analysis

The transient expression of *T. hemsleyanum* was used to determine superoxide dismutase (SOD, EC 1.15.1.1), peroxidase (POD, EC 1.11.1.7) activity, and malondialdehyde (MDA) content. POD activity was determined as guaiacol oxidation by H_2_O_2_. SOD activity was analyzed based on the inhibiting rates of the reduction of nitro blue tetrazolium (NBT), whereas MDA content was extracted and determined using the thiobarbituric acid reaction method according to our previous report (Peng et al., 2019). Proline content was determined using a colorimetric method (Bates et al., 1973). Leaf electrolyte leakage (EL) was calculated based on the formula (%) = C_initial_/C_max_ × 100, where C_initial_ indicates the initial conductivity and C_max_ represents the max conductivity. Fresh leaves (0.2 g) were soaked instantly in 50 ml of distilled water for 24 h to detect the C−initial. These samples were autoclaved at 120°C for 20 min and cooled down to room temperature to detect the C−max using a conductivity meter (YSI Model 32, Yellow Spring, OH, United States) (Blum and Ebercon, 1981). Evans blue was used to detect the activity of leaf cells in different treatments according to our previous study (Xie et al., 2022). In brief, the leaves were immersed in an aqueous solution of 1 mg⋅ml^–1^ Evans blue and incubated for 12 h in the dark. The leaves were set to the ethanol:lactic acid:glycerol (3:1:1) mixture to boil for 5 min and stored in 60% glycerol before photography.

## Results

### Identification, physicochemical properties, and subcellular localization prediction of *Tetrastigma hemsleyanum*’s AP2/ERF transcription factors

A total of 104 ThERF sequences were identified in the *T. hemsleyanum* transcriptome. These sequences were first annotated using the NR and Swiss-Prot databases, then they were identified and analyzed using the NCBI-CDD online website. Finally, 70 ThERF sequences were obtained. The physicochemical properties of the proteins encoded by the ThERF TF family were predicted. The results indicated that the protein length, molecular weight, and theoretical isoelectric point of ThERF TFs were 128–684 aa, 13.93–76.07 kD, and 4.34–9.85, respectively. The instability coefficients were in the range of 32.72–81.44, with 94.28% of the proteins being unstable proteins with an instability coefficient of over 40. The average hydrophilic coefficients were lower than 0, and all ThERFs were hydrophilic proteins. The results of the subcellular localization prediction suggested that 52 ThERF TFs were located in the nucleus, accounting for 74.28%; 10 ThERF TFs were located in the cytoplasm, accounting for 14.28%; and 7 ThERF TFs were located in the cytoplasm and nucleus, accounting for 10%. Notably, ThERF5 was located in the cytoplasm and mitochondria (Table 1). The ThERF proteins were mainly composed of acidic and unstable hydrophilic proteins, which were primarily expressed in the nucleus.

**TABLE 1:** The AP2/ERF protein information of *T. hemsleyanum*.

Gene ID	Gene name	Protein length(aa)	Molecular weight (kD)	pI	Instability index	Grand average of hydropathicity	Subcellular location prediction
*CL10332.Contig1_All*	*ThERF1*	429	45.11	5.58	71.33	–0.534	Nucleus
*CL10442.Contig1_All*	*ThERF2*	347	38.67	4.96	52.31	–0.647	Cytoplasm, Nucleus
*CL11106.Contig1_All*	*ThERF3*	219	23.80	6.6	59.37	–0.489	Nucleus
*CL11236.Contig1_All*	*ThERF4*	385	42.32	5.57	64.92	–0.527	Nucleus
*CL11946.Contig3_All*	*ThERF5*	368	41.57	6.06	47.51	–0.731	Cytoplasm, Mitochondrion
*CL1405.Contig1_All*	*ThERF6*	457	51.05	6.11	54.06	–0.809	Nucleus
*CL166.Contig7_All*	*ThERF7*	277	30.37	8.31	61.01	–0.812	Nucleus
*CL1944.Contig1_All*	*ThERF8*	323	36.53	4.92	64.25	–0.785	Cytoplasm, Nucleus
*CL2218.Contig2_All*	*ThERF9*	160	18.11	6.92	61.55	–0.547	Nucleus
*CL2330.Contig2_All*	*ThERF10*	346	39.55	8.33	64.53	–0.777	Nucleus
*CL2397.Contig1_All*	*ThERF11*	265	29.69	5.43	58.89	–0.505	Cytoplasm. Nucleus
*CL2883.Contig1_All*	*ThERF12*	179	20.28	8.77	57.11	–0.817	Cytoplasm
*CL3152.Contig1_All*	*ThERF13*	235	26.28	5.78	62.36	–0.803	Nucleus
*CL3936.Contig3_All*	*ThERF14*	491	56.29	5.82	45.17	–0.709	Cytoplasm, Nucleus
*CL4901.Contig1_All*	*ThERF15*	240	25.82	9.85	81.44	–0.432	Nucleus
*CL503.Contig11_All*	*ThERF16*	500	54.87	6.81	59.51	–0.64	Cytoplasm
*CL5075.Contig6_All*	*ThERF17*	463	52.35	8.1	61.83	–0.82	Nucleus
*CL6261.Contig1_All*	*ThERF18*	358	39.19	5.03	48.01	–0.626	Nucleus
*CL6488.Contig1_All*	*ThERF19*	256	27.66	5.02	58.25	–0.454	Nucleus
*CL8075.Contig1_All*	*ThERF20*	313	34.90	5.79	35.32	–0.809	Nucleus
*CL8199.Contig1_All*	*ThERF21*	206	22.64	4.7	45.09	–0.457	Cytoplasm, Nucleus
*CL9634.Contig2_All*	*ThERF22*	369	41.00	5.01	39.18	–0.894	Nucleus
*Unigene14424_All*	*ThERF23*	337	37.71	4.34	58.6	–0.575	Nucleus
*Unigene14425_All*	*ThERF24*	339	38.24	4.78	61.93	–0.755	Nucleus
*Unigene14760_All*	*ThERF25*	231	25.72	7.96	72.93	–0.897	Nucleus
*Unigene14895_All*	*ThERF26*	234	25.64	8.86	51.4	–0.5	Nucleus
*Unigene15011_All*	*ThERF27*	226	24.26	4.71	66.57	–0.56	Cytoplasm, Nucleus
*Unigene1509_All*	*ThERF28*	181	20.19	4.89	57.32	–0.772	Cytoplasm
*Unigene15444_All*	*ThERF29*	230	24.83	5.85	70.85	–0.391	Cytoplasm
*Unigene16789_All*	*ThERF30*	456	51.30	6.89	63.02	–0.783	Nucleus
*Unigene17167_All*	*ThERF31*	203	22.47	8.76	71.52	–0.923	Nucleus
*Unigene17341_All*	*ThERF32*	147	16.30	6.15	46.33	–0.912	Nucleus
*Unigene17356_All*	*ThERF33*	208	23.59	5.3	61.33	–0.597	Nucleus
*Unigene17420_All*	*ThERF34*	245	26.37	8.89	56.78	–0.389	Nucleus
*Unigene17549_All*	*ThERF35*	128	13.93	5.92	47.93	–0.372	Cytoplasm
*Unigene17818_All*	*ThERF36*	168	18.41	9.64	56.95	–0.579	Nucleus
*Unigene19316_All*	*ThERF37*	363	40.01	9.48	44.62	–0.749	Nucleus
*Unigene19512_All*	*ThERF38*	317	33.80	6.09	55.2	–0.452	Nucleus
*Unigene20038_All*	*ThERF39*	340	38.04	5.46	52.72	–0.641	Nucleus
*Unigene20053_All*	*ThERF40*	285	31.72	5.25	66.88	–0.568	Nucleus
*Unigene21865_All*	*ThERF41*	324	35.91	4.86	54.13	–0.622	Nucleus
*Unigene22413_All*	*ThERF42*	253	27.82	5.24	61	–0.66	Cytoplasm
*Unigene22539_All*	*ThERF43*	214	24.10	5.12	61.95	–0.496	Nucleus
*Unigene23417_All*	*ThERF44*	194	21.49	5.55	60.6	–0.626	Nucleus
*Unigene2351_All*	*ThERF45*	182	19.79	5.39	53.39	–0.449	Cytoplasm, Nucleus
*Unigene24800_All*	*ThERF46*	170	18.97	9.73	49.06	–0.744	Nucleus
*Unigene24813_All*	*ThERF47*	245	27.48	6.25	63.1	–0.647	Nucleus
*Unigene24879_All*	*ThERF48*	288	30.74	6.61	66.15	–0.41	Nucleus
*Unigene25069_All*	*ThERF49*	227	24.16	9.51	56.62	–0.357	Nucleus
*Unigene25131_All*	*ThERF50*	469	51.86	5.34	67.61	–0.874	Nucleus
*Unigene25231_All*	*ThERF51*	144	16.38	8.77	48.27	–0.953	Nucleus
*Unigene25415_All*	*ThERF52*	456	51.30	6.89	63.02	–0.783	Nucleus
*Unigene27354_All*	*ThERF53*	267	30.23	9.35	73.57	–1.017	Nucleus
*Unigene27504_All*	*ThERF54*	221	24.19	5.16	44.28	–0.511	Cytoplasm
*Unigene27991_All*	*ThERF55*	148	16.53	6.29	44.8	–0.464	Nucleus
*Unigene29462_All*	*ThERF56*	254	27.33	4.9	76.98	–0.555	Nucleus
*Unigene29470_All*	*ThERF57*	365	40.90	9.4	47.89	–0.715	Nucleus
*Unigene29712_All*	*ThERF58*	331	35.70	9.2	43.19	–0.564	Nucleus
*Unigene29897_All*	*ThERF59*	189	20.54	9.6	63.12	–0.497	Nucleus
*Unigene300_All*	*ThERF60*	262	29.57	5.4	56.45	–0.699	Nucleus
*Unigene30691_All*	*ThERF61*	138	15.01	9.74	38.12	–0.851	Cytoplasm
*Unigene32298_All*	*ThERF62*	255	27.82	6.74	32.72	–0.638	Nucleus
*Unigene32510_All*	*ThERF63*	173	19.57	9.82	71.39	–0.934	Nucleus
*Unigene32524_All*	*ThERF64*	684	76.07	6.57	59.39	–0.748	Nucleus
*Unigene32553_All*	*ThERF65*	241	27.41	6.4	58.02	–0.637	Cytoplasm
*Unigene34083_All*	*ThERF66*	684	76.07	6.57	59.39	–0.748	Nucleus
*Unigene3711_All*	*ThERF67*	368	41.07	9.14	46.88	–0.583	Nucleus
*Unigene755_All*	*ThERF68*	242	25.96	4.91	59.79	–0.648	Cytoplasm
*Unigene774_All*	*ThERF69*	234	24.98	5.8	46.39	–0.613	Nucleus
*Unigene958_All*	*ThERF70*	371	40.66	6.01	58.97	–0.616	Nucleus

### Identification of the conserved motifs of *Tetrastigma hemsleyanum*’s AP2/ERF transcription factors

**FIGURE 1:**
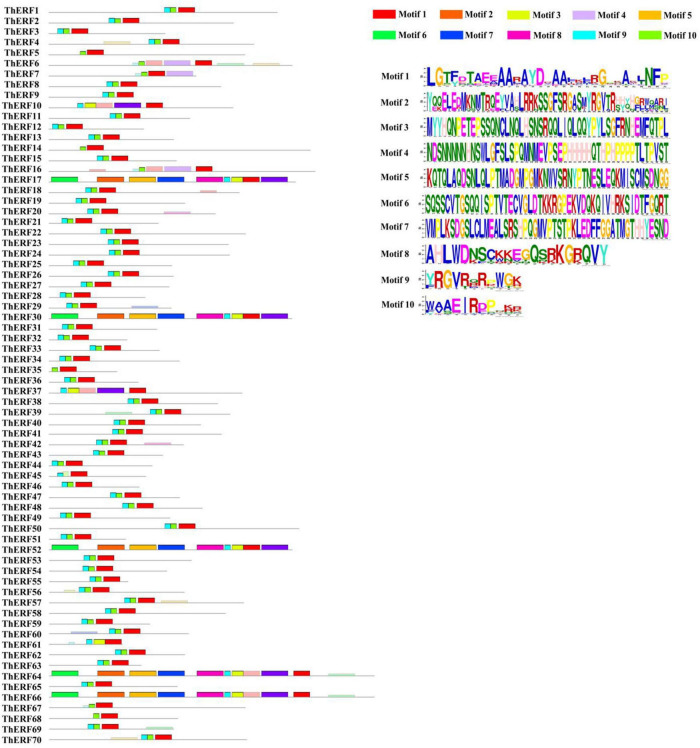
Distribution of conserved motifs of the AP2/ERF transcription factors in *T. hemsleyanum*. Different motifs are highlighted in different colors.

Multiple sequence alignments and conserved domains of AP2/ERF proteins were identified using the DNAMAN software and NCBI CD search online tools, respectively (Supplementary Figure S1). All AP2/ERF proteins were divided into cl00033 (95.71%), cl15242 (2.85%), and cl11268 (1.42%). Cl00033 was located at the C-terminal of the ThERF8 protein domain (Supplementary Table S2). The conserved motifs of 70 AP2/ERF proteins were analyzed using the MEME online tool. A total of 10 conserved motifs were detected (Figure 1). Motif 1, motif 2, and motif 3 located all AP2/ERF proteins (100%). Motif 4 to motif 10 existed mainly in ThERF17, ThERF30, ThERF52, ThERF64, and ThERF66. Motif 4 was also identified in ThERF6, ThERF7, ThERF10, ThERF16, and ThERF37. Motif 4 to motif 10 were unavailable in AP2/ERF proteins, suggesting that they may have been caused by the long evolution of *T. hemsleyanum*.

### Phylogeny of *Tetrastigma hemsleyanum*’s AP2/ERF transcription factors

**FIGURE 2:**
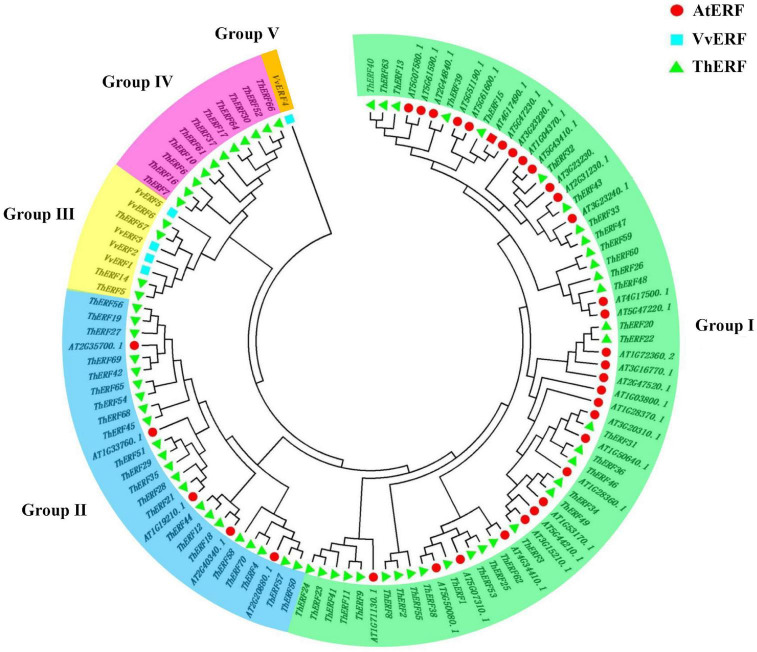
Phylogenetic tree of AP2/ERF transcription factors in *T. hemsleyanum*. Deduced full-length amino acid sequences were used to construct the phylogenetic tree with the MEGA 7.0 software using a neighbor-joining method with 1,000 bootstrap replicates. Five groups are highlighted in different colors.

To confirm the classification and evolutionary relationships of AP2/ERF TFs in *T. hemsleyanum*, the full-length sequences of the putative proteins were compared, and a phylogenetic tree analysis was conducted. All AP2/ERF proteins could be classified into five groups (Figure 2). Group I included 34 TFs, containing motif 1, motif 6, and motif 9. Group II included 22 TFs, almost all of which contained motif 1 and motif 6. Motif 9 was not found in ThERF35, ThERF45, and ThERF68. Groups I and II were considered members of the DREB subfamily. A large proportion of the TFs in these groups containing two AP2 domains was classified into the AP2 family. Group III, similar to the second group, contained 3 TFs. ThERF5 and ThERF67 included motif 1 and motif 6 but had lost motif 9. Most TFs in this group were classified into the RAV family. Group IV included 11 TFs, and almost all ThERF TFs contained motif 4 and motif 8. The TFs, including ThERF7, ThERF30, ThERF64, and ThERF66 in other clades, contained almost all identified motifs.

### Three-dimensional structure models of *Tetrastigma hemsleyanum*’s AP2/ERF transcription factors

Swiss-model software was used to predict three-dimensional (3D) structure models of key ThERF TFs (Supplementary Figure S2). As a result, ThERFs could be divided into four types of structures, and the identified similarity of all sequences ranged from 36.99 to 98.53. Group I consisted of 10 ThERF proteins, including ThERF31, ThERF32, ThERF40, ThERF44, ThERF45, ThERF46, ThERF48, ThERF49, ThERF55, and ThERF56, which had been identified as GCC-box containing, ethylene-responsive TFs. Group II and group III included ThERF5 and ThERF67, and ThERF35 and ThERF37 with similar 3D models, which were identified as the DNA-binding protein RAV1 solution structures of the B3 DNA-binding domain of RAV1 and a B3 domain-containing transcription reporter, respectively. Group IV contained ThERF14, which was described as a NAD (P) H-quinone oxidoreductase subunit M chloroplast NDH complex special structure (Supplementary Table S3). The results indicated that these ThERF proteins had different functions in the growth and development of *T. hemsleyanum*.

### Expression profiling of *AP2/ERF* genes under cold stress

**FIGURE 3:**
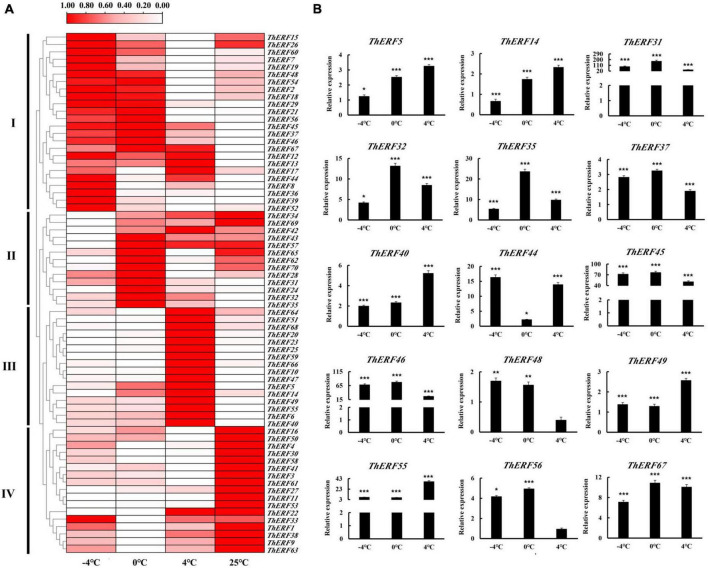
Expression profiles of AP2/ERF TFs at (–4, 0, and 4°C) cold treatments. **(A)** Heat map for gene expression of 70 *ThERFs* in *T. hemsleyanum* under cold treatments. **(B)** Gene expression of 15 key *ThERFs* in *T. hemsleyanum* under cold treatments. The heat map is constructed based on the Log2 (relative expression level of each transcription factor) detected by qRT-PCR. The color scale represents the Log2 (relative expression level of each transcription factor), with white denoting low expression and red denoting high expression. Data are means ± standard error of the three biological replicates for each treatment. *P ≤ 0.05, **P ≤ 0.01, ***P ≤ 0.001.

Numerous studies suggested that the AP2/ERF family plays an important role in plants’ resistance to cold stress. To prove this conjecture, the expression profiles of AP2/ERF TFs were explored under different cold stress conditions for 6 h. All 70 *ThERFs* were shown to have different expression levels under cold stress (Supplementary Figures S3–S5). To facilitate the observation, the expression levels of all *ThERFs* in *T. hemsleyanum* were described as a heat map (Figure 3A). In addition, the 15 key *ThERFs*, including *ThERF5*, *ThERF14*, *ThERF31*, *ThERF32*, *ThERF35*, *ThERF37*, *ThERF40*, *ThERF44*, *ThERF45*, *ThERF46*, *ThERF48*, *ThERF49*, *ThERF55*, *ThERF56*, and *ThERF67* were selected for detailed analysis (Figure 3B). A total of 13 *ThERFs* increased 1.89-fold to 68.4-fold compared with the control (*P* < 0.01), while the relative expression levels of *ThERF48* and *ThERF56* decreased 0.39-fold and 0.94-fold compared with the control, respectively. The expression levels of 15 key *ThERFs* increased significantly at 0 and -4°C, increasing 1.28-fold to 174.57-fold and 1.37-fold to 90.57-fold compared with the 25°C treatment, respectively (*P* < 0.01). The findings suggested that these key *ThERFs* were significantly influenced by cold stress and played an important role in the resistance to cold stress.

### Identification of the functions of *ThERF46* in *Tetrastigma hemsleyanum* under cold stress

**FIGURE 4:**
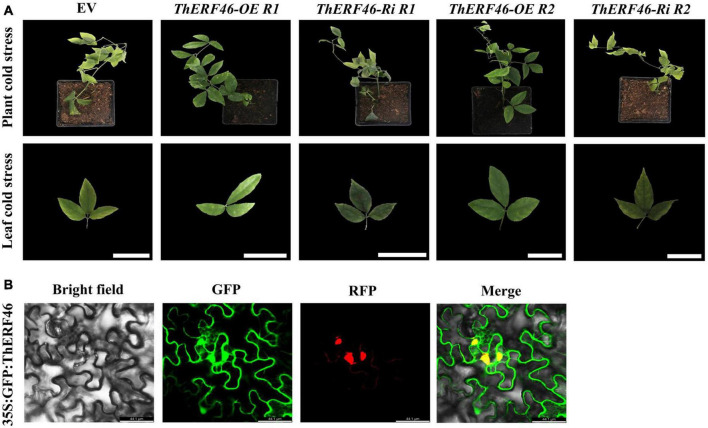
Phenotype and subcellular localization analysis of ThERF46 in *T. hemsleyanum.* **(A)** The phenotype of *ThERF46*-OE, *ThERF46*-Ri, and EV treatment under cold stress. EV, empty vector (the EV without an exogenous gene was used as the control); R1, Repeat 1; R2, Repeat 2. **(B)** Subcellular localization of ThERF46 in *N. benthamiana.* GFP, green fluorescence protein; RFP, red fluorescence protein (used as a marker of nucleus localization signals). Three biological replicates for each treatment. Bar, 0.5 cm.

**FIGURE 5:**
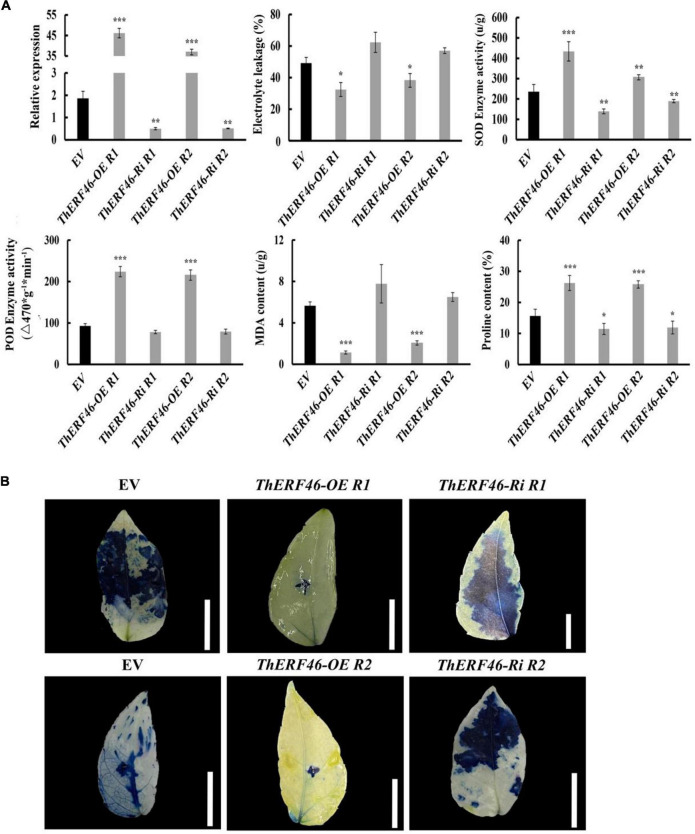
Analysis of the biochemical indexes of *ThERF46* in *T. hemsleyanum.* **(A)** Analysis of the physiological indexes of *T. hemsleyanum*. **(B)** Analysis of cell activity of *T. hemsleyanum*. EV, empty vector (the EV without an exogenous gene was used as the control); R1, Repeat 1; R2, Repeat 2. Data are means ± standard error of the three biological replicates for each treatment. Bar, 1 cm. *P ≤ 0.05, **P ≤ 0.01, ***P ≤ 0.001.

To further determine the biological function of *ThERF*s, the ORFs of 15 key *ThERFs* were predicted, and the complete CDS sequence of *ThERF46* was cloned in overexpression and RNA-interference expression vectors. The transient *ThERF46*-OE and *ThERF46*-Ri leaves were subjected to cold stress for 6 h. After 6 h of 4°C cold treatment, it can be clearly found that the leaves of the empty vector (EV) and *ThERF46*-Ri treatment had water stains and wilted, and could not extend completely under cold stress by phenotypic observation. In addition, after transient overexpression of *ThERF46*, the leaves did not show obvious damage from cold stress. The *ThERF46*-Ri and EV leaves were damaged more severely than *ThERF46*-OE leaves (Figure 4A). Subcellular localization analysis found that ThERF46 was located in the nucleus and cell membrane of *N. benthamiana* (Figure 4B). The expression levels of *ThERF46*-OE and *ThERF46*-Ri were significantly increased and decreased 46-fold and 0.3-fold compared with the EV leaves, respectively. Under cold stress conditions, the MDA content and relative electrolyte leakage of *ThERF46*-OE and *ThERF46*-Ri leaves were quantified. The resulting MDA content and relative electrolyte leakage leaves were significantly decreased to 1.6 ± 3.28% and 31.2 ± 6.32% in *ThERF46*-OE compared with EV, respectively. The SOD enzyme activity, POD enzyme activity, and proline content showed a dramatic increase to 421.4 ± 12.3%, 223.8 ± 11.2%, and 27.3 ± 9.1%, respectively (Figure 5A). Moreover, the results also showed there was a higher content of proline and less damage area in *ThERF46*-OE leaves compared with EV and *ThERF46*-Ri treatments (Figure 5B). These results showed that the transient overexpression of *ThERF46* reduced the cell membrane damage and positively regulated cold tolerance by enhancing the activity of the plant cell and antioxidant enzyme system of *T. hemsleyanum* under cold stress.

## Discussion

In contrast to animals, plants must be subjected to different biological and abiotic stresses during their growth and development, such as temperature, light, precipitation, humidity, and soil conditions. Among them, cold stress is the key factor affecting plant growth and development (Chinnusamy et al., 2010). The special growth requirements of *T. hemsleyanum* are an important problem challenge for the artificial cultivation industry. The quality and quantity of *T. hemsleyanum* have always suffered from freezing damage. Response to cold stress is a very complex process in plants, involving various pathways, metabolic pathways, gene expression, intercellular changes, cold signal transduction, gene transcription, and post-translational alterations in plants (Chinnusamy et al., 2010). Several mechanisms would minimize the potential damage caused by cold stress in plants, involving a series of physiological and biochemical modifications (Guo et al., 2021). During this period, the expression of many genes, proteins, and metabolites in plants would also change in response to cold stress (Guo et al., 2021). Therefore, it is important to further study the response mechanism of *T. hemsleyanum* under cold stress.

AP2/ERF TFs are one of the most specific and largest TF families in plants (Rashid et al., 2012; Licausi et al., 2013). They not only participate in the regulation of plant growth and development but also play an important role in plant stress. In recent years, with the development of sequencing technology in plant genomes, the distribution of AP2/ERF TFs in different plants has been gradually revealed. The AP2/ERF TF subfamily has also been studied extensively in many plants. More than 100 AP2/ERF TFs have been identified and investigated in many plants, but there has not been a relevant investigation of the AP2/ERF family in *T. hemsleyanum* yet (Zhuang et al., 2008, 2011; Licausi et al., 2010; Sharoni et al., 2011; Pirrello et al., 2012; Song et al., 2013; Shu et al., 2016). In this study, 70 AP2/ERF TFs were identified in *T. hemsleyanum*, which were fewer than those identified in *Vitis vinifera* (149). This may be due to long-term evolutionary relationships or maybe an inevitable factor caused by transcriptome data. A further phylogenetic tree analysis of AP2/ERF among *T. hemsleyanum*, *Vitis vinifera*, and *Arabidopsis* indicated that the number of TFs in the AP2 family and RAV and DERB subfamilies in *T. hemsleyanum* was significantly lower than that in *Vitis vinifera* and *Arabidopsis*. The findings suggested that the difference in the number of AP2/ERF TFs in *T. hemsleyanum* was mainly caused by the contractions of AP2 and DREB TFs and the amplification of some ERF TFs (Li et al., 2017). *VvERF4* in *Vitis vinifera* was classified into a single group, indicating a long evolutionary distance of genetic relationships between *T. hemsleyanum* and *Vitis vinifera* TFs, which may be the result of a long evolution.

In the phylogenetic analysis, a few ThERF TFs were identified in group I, which included most AtERF TFs. ThERF37, ThERF45, ThERF56, and ThERF67 in *T. hemsleyanum* differed at the evolutionary level but also showed similar expression changes, suggesting that they had the same functions under cold stress (Licausi et al., 2010; Zhang et al., 2022). In general, the domains or amino acid motifs of TFs are often involved in nuclear localization, DNA-binding, protein-protein interaction, and transcriptional activity (Nakano et al., 2006). The B3 domain in RAV TFs played a vital role in abiotic stress and pathogen resistance (Kim et al., 2005; Cao et al., 2020; Cui et al., 2021; Zhang et al., 2021). The core sequence of the B3 special domain was identified in ThERF5, ThERF35, and ThERF67, which belonged to the RAV TF family. The RAV (B3) special domains were confirmed to be a transcriptional repressor in plants (Hu et al., 2004; Mittal et al., 2015). The qRT-PCR results showed that cold stress activated the expression of ThERF5, ThERF35, and ThERF67, which contained a B3 domain. These findings indicate that a function of the B3 domain in ThERFs under cold stress should be further explored. In addition, most AP2/ERF TFs with an AP2 domain in *T. hemsleyanum* were specifically expressed at high levels during cold stress resistance, suggesting that they played the same functions as the B3 domains. Some domains or motifs of ThERFs were highly conserved, and the newly evolved motifs had been generated in the long evolutionary process of *T. hemsleyanum*, which might play an important role in the subfunctionalization or new functions of AP2/ERF TFs in specific plant species. Notably, the Ndhm domain, which was annotated as a medium for electron transfer during the process of PS I in vascular plants, was identified in ThERF14 (Yamamoto et al., 2016). The Ndhm domains were not common in the AP2/ERF TF family. This may be a specific domain in the AP2/ERF TF family in *T. hemsleyanum*. The function and regulation of these newly evolved motifs in AP2/ERF TFs should be further explored, and this study provides important insights for further explaining the evolution and functions of AP2/ERF TFs.

To further study the potential mechanisms of AP2/ERF TFs in response to cold stress, the expression patterns of 70 AP2/ERF TFs were analyzed. Almost all 70 AP2/ERF TFs were activated under cold stress, and the 15 key *ThERFs* rapidly responded to the beginning cold stress. These findings are in line with previous reports on the involvement of AP2/ERF TFs in abiotic stress (Mizoi et al., 2012; Velivelli et al., 2015; Shu et al., 2016). Then, the relevant resistance genes were expressed at high levels to resist cold stress during the intermediate stages of cold stress processes. Eleven key AP2/ERF proteins, including ThERF31, ThERF32, ThERF37, ThERF40, ThERF44, ThERF45, ThERF46, ThERF48, ThERF49, ThERF55, and ThERF56, showed the same expression profiles under these conditions. These findings indicate that *ThERFs* play an important role in the tolerance to cold stress in *T. hemsleyanum*, and their role should be explored further. Then, with stress exacerbation, plants could not resist the stress only *via* their own regulation, resulting in metabolic disorder, cell activity, and decreased gene expression (Fowler and Thomashow, 2002; Dong and Liu, 2010). During this period, the expression levels of *ThERFs* also showed a downward trend compared to the initial stage of cold stress (Figure 3B).

Plant cell tissue is the primary system to respond to cold stress, and cold stress affects the membrane of plant cells, which is considered to be the main reaction to trigger the cold stress response in plants (Orvar et al., 2000). The membrane system changes mainly in membrane lipid transformation and membrane lipid peroxidation under cold stress conditions (Ye et al., 2019; Zhuang et al., 2019). Membrane permeability is also an important physiological index to evaluate cold resistance during membrane lipid transformation. The electrolyte leakage was negatively correlated with plant resistance under cold stress. In the process of membrane lipid peroxidation, the activity of SOD and POD enzymes is rapidly induced under cold stress and decreases the accumulation of H_2_O_2_ and ROS in plant cells (Theocharis et al., 2012). In addition, MDA is the final product of lipid peroxidation, and over-accumulation of MDA can change the structure of the cell membrane and aggravate membrane lipid phase transformation and membrane damage (Huang et al., 2017; Yu et al., 2018; Peng et al., 2019). Proline is very hydrophilic. It can stabilize the protoplast colloid and the metabolic process and reduce the chilling injury to prevent cell dehydration (Guo et al., 2021). Therefore, the content of these molecules and enzyme activity are widely used to evaluate the resistance of plants under cold stress. Gene function analysis was considered an important approach to understanding the molecular mechanisms of plant response to abiotic stress. In this study, *ThERF46* was identified and cloned, and subcellular localization determined its functional location. The detection of physiological functions under cold stress confirmed the role of *ThERF46* in the resistance of *T. hemsleyanum*. Transient overexpression of *ThERF46* increased the tolerance by enhancing the activity of cell and antioxidant enzyme systems of *T. hemsleyanum*, while transient RNA-interference expression of *ThERF46* decreased the tolerance of cold stress in *T. hemsleyanum*. These results indicate that *ThERF46* plays an important role in *T. hemsleyanum* during the cold stress process. Nevertheless, although several AP2/ERF TFs have been proven to play an important role in the tolerance of plants to various biological and abiotic stresses, the function of AP2/ERF TFs in *T. hemsleyanum* is still largely unknown. This study identified potential candidate genes for the AP2/ERF TFs, especially *ThERF46*, to further explore their role in *T. hemsleyanum* under cold stress.

## Conclusion

In conclusion, 70 AP2/ERF TFs were identified in *T. hemsleyanum*. Clustering and phylogenetic analyses were conducted to divide these TFs into five groups. Ten conserved motifs were identified in the 70 AP2/ERF TFs. AP2/ERF TFs exhibiting specific expression patterns under cold stress were also confirmed. Transient overexpression and RNA-interference expression of *ThERF46* increased and decreased the tolerance to cold stress, respectively. These findings provide new insights into the AP2/ERF TFs in *T. hemsleyanum* and identify candidate AP2/ERF TFs to further elucidate their roles in cold stress tolerance.

## Data availability statement

The transcriptome datasets presented in this study can be found in the NCBI database (PRJNA797653).

## Author contributions

ZZ and XP conceived the study. ML designed the experiment. ZX performed the physiological indicator determination and expression analysis. CY and LG performed the RNA extraction and quality determination. SL carried out the analysis. All authors have read and approved the manuscript.

## Footnotes

1
http://pfam.Xfam.org/

2
http://meme-suite.org/tools/meme

3
http://arabidopsis.org

4
https://wolfpsort.hgc.jp/

5
https://web.expasy.org/protparam/

6
https://swissmodel.expasy.org/

## Supplementary material

Supplementary Figure 1 Multiple sequence alignments of AP2/ERF TFs in *T. hemsleyanum*.

Supplementary Figure 2 Protein 3D model of AP2/ERF transcription factors in *T. hemsleyanum*. Full-length amino acid sequences were used to construct the 3D model (similarity model > 30%).

Supplementary Figure 3 Gene expression of ThERF1–ThERF25 in *T. hemsleyanum* under cold stress.

Supplementary Figure 4 Gene expression of ThERF26–ThERF50 in *T. hemsleyanum* under cold stress.

Supplementary Figure 5 Gene expression of ThERF51–ThERF70 in *T. hemsleyanum* under cold stress.
