# Supplementary figures and images for: Identification of AP2/ERF transcription factors in Tetrastigma hemsleyanum revealed the specific roles of ERF46 under cold stress
Source: Front Plant Sci. 2022 Aug 9;13:936602. doi: 10.3389/fpls.2022.936602 (PMC9396264; doi:10.3389/fpls.2022.936602)

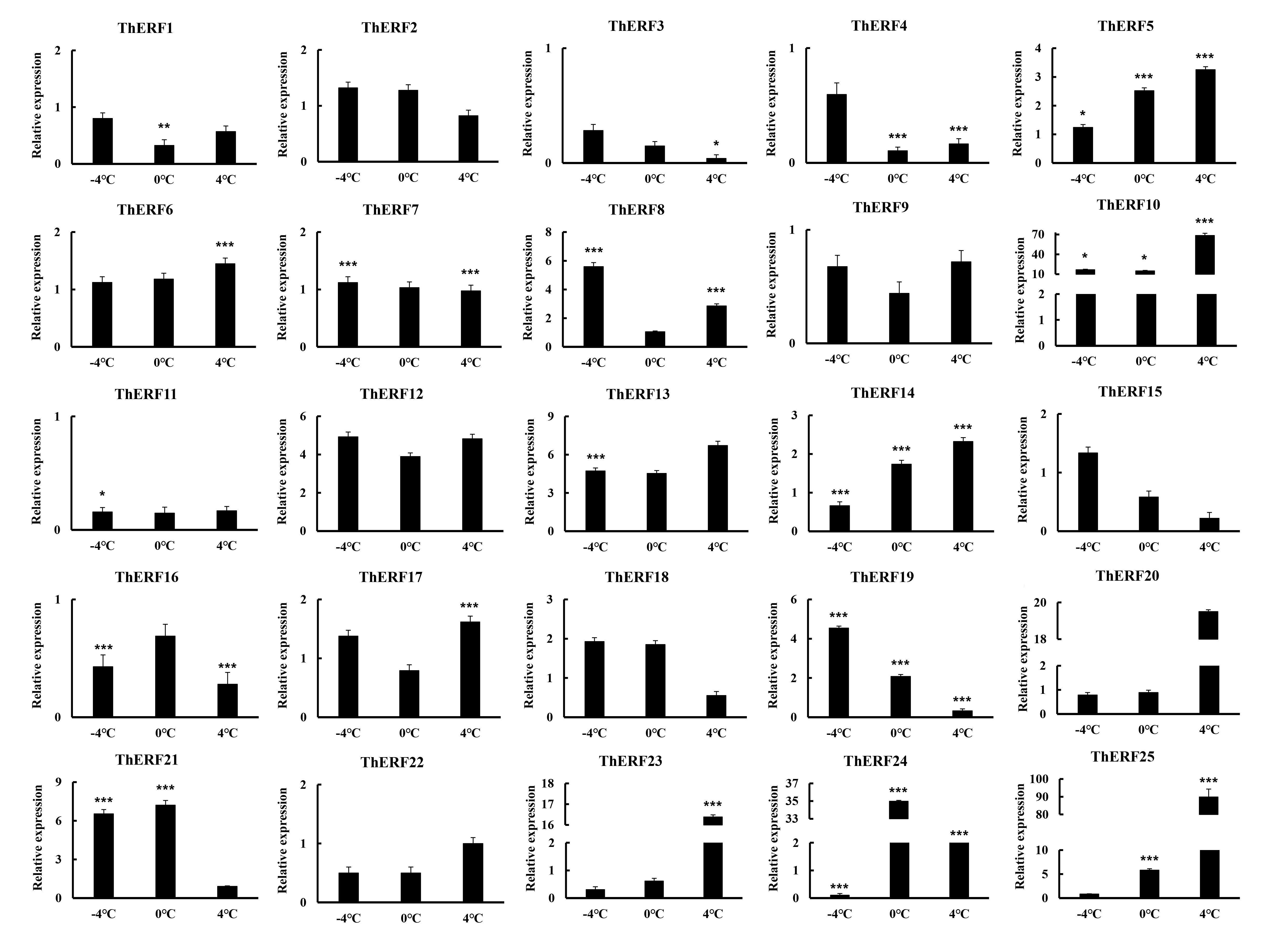

Supplement: Supplementary Figure 3 — Gene expression of ThERF1–ThERF25 in T. hemsleyanum under cold stress. [file Image_3.JPEG]

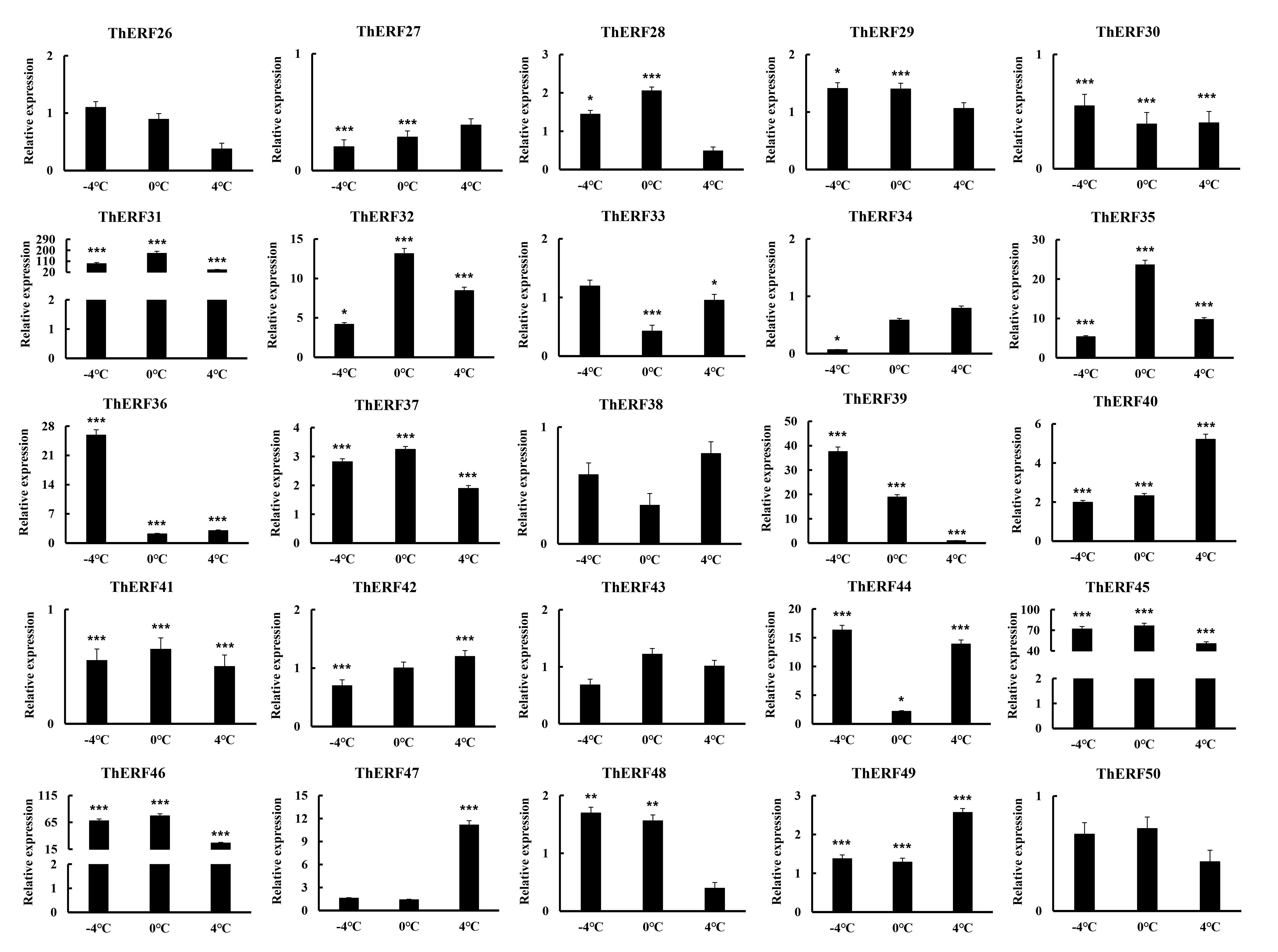

Supplement: Supplementary Figure 4 — Gene expression of ThERF26–ThERF50 in T. hemsleyanum under cold stress. [file Image_4.JPEG]

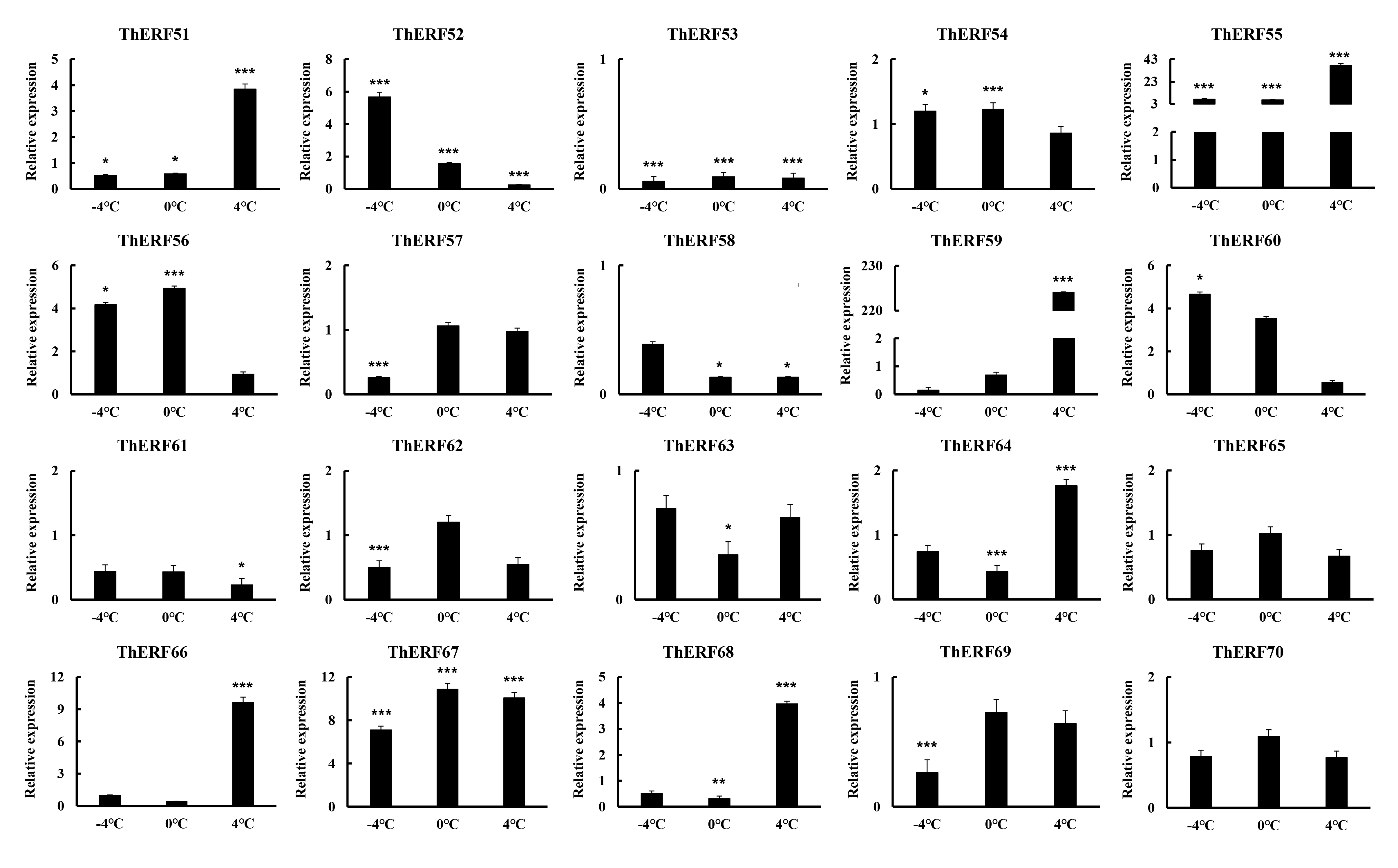

Supplement: Supplementary Figure 5 — Gene expression of ThERF51–ThERF70 in T. hemsleyanum under cold stress. [file Image_5.JPEG]
