# Supplementary material for: Identification of AP2/ERF transcription factors in Tetrastigma hemsleyanum revealed the specific roles of ERF46 under cold stress
Source: Front Plant Sci. 2022 Aug 9;13:936602. doi: 10.3389/fpls.2022.936602 (PMC9396264; doi:10.3389/fpls.2022.936602)
Supplement: Supplementary file 6 [file Table_1.DOCX]

Table. S1 Primers used in qRT-PCR assay.

| Gene ID | Gene Name | Primer F (5’-3’) | Primer R (5’-3’) | Tm | Length(bp) |
| --- | --- | --- | --- | --- | --- |
| CL10332.Contig1_All | ThERF1 | CGACATTACGAGAGACTACT | ATGGAGATGGAGATGAAGAG | 60 | 158 |
| CL10442.Contig1_All | ThERF2 | TAAAGGAGTTCGCCGTAG | CTGGTGACTGCTTATTCTTC | 60 | 188 |
| CL11106.Contig1_All | ThERF3 | CAAAGAGCCACATTTCCG | GGAGAGGGAAGTTGGTTT | 60 | 188 |
| CL11236.Contig1_All | ThERF4 | ATACAGTAGCAGCAGGAC | GAAGGAAGAAGAGGAGAAGT | 60 | 168 |
| CL11946.Contig3_All | ThERF5 | GGCTTATGAGAGGAACACT | CTACCACAGACTGAGACTC | 60 | 176 |
| CL1405.Contig1_All | ThERF6 | CGACAACGAACTCAACTC | GTCATCCCAACTGAATGC | 60 | 144 |
| CL166.Contig7_All | ThERF7 | GATGACGACTCTTGCTCTA | GAATCTCCACCAACTCCT | 60 | 146 |
| CL1944.Contig1_All | ThERF8 | ATGTCTCTTCCTCGGTTG | GCGTGGATAAGATTGGTTC | 60 | 193 |
| CL2218.Contig2_All | ThERF9 | TTAGGCGAAGTCGGTATG | TGAATGATCCTCACTCTCC | 60 | 173 |
| CL2330.Contig2_All | ThERF10 | GCTACAACTGTGAAGAGAAG | CATCATAGGCTCCAAGGT | 60 | 148 |
| CL2397.Contig1_All | ThERF11 | TTAGGCGAAGTCGGTATG | TGAATGATCCTCACTCTCC | 60 | 173 |
| CL2883.Contig1_All | ThERF12 | CACGACTCACACTCATCT | CATCTCTGCCCAAATTCTG | 60 | 154 |
| CL3152.Contig1_All | ThERF13 | TTCTACTTCTTCTCCGTCTG | AGGAACTGAGATGCTGAG | 60 | 100 |
| CL3936.Contig3_All | ThERF14 | CGTGGCTTATGAGAGGAA | GGTTCTACTGGTCTTGGT | 60 | 198 |
| CL4901.Contig1_All | ThERF15 | TTTCCTCCGTCTCTCCTA | TCTGTAGTGCCTCTTGTC | 60 | 116 |
| CL503.Contig11_All | ThERF16 | GGCGATGAGGATTCTTCTA | CACTGAGGCTTTGGAAAC | 60 | 200 |
| CL5075.Contig6_All | ThERF17 | GGCAGCACTCAAGTATTG | CTCCTGTCTGGTCATGTT | 60 | 100 |
| CL6261.Contig1_All | ThERF18 | AGAGGTAGTAGGCTATGGT | GGTTGGAACTGAGGAAGA | 60 | 153 |
| CL6488.Contig1_All | ThERF19 | TCGTTGACTCAGTAGATGG | GGTTGGAATCAGGATCTCT | 60 | 119 |
| CL8075.Contig1_All | ThERF20 | CTCCAATGGCTTCAACAC | CATTCCAGACGAAGAGTAGA | 60 | 109 |
| CL8199.Contig1_All | ThERF21 | CATAGGCTCCGACACTTAT | CTGCGTAGTCATTTGTAGAG | 60 | 104 |
| CL9634.Contig2_All | ThERF22 | CATCATCTCCGACTTCATTC | AGTCTTCGTCATCCTCTTC | 60 | 119 |
| Unigene14424_All | ThERF23 | CGACGAACACAGAAGATTC | CCGAAGTTTCCTCAAAGATC | 60 | 111 |
| Unigene14425_All | ThERF24 | GGAAGAAGTTCAGAGGAGT | CGTTGTCGTACACCATTG | 60 | 135 |
| Unigene14760_All | ThERF25 | AGGAGACGACACTACAGA | CAGGGAAGTTGAGTTTAGC | 60 | 184 |
| Unigene14895_All | ThERF26 | GTGTTTGACGGAGAACTG | TCGCTACTTCTTCCATAGAC | 60 | 158 |
| Unigene15011_All | ThERF27 | GTCTTCAACCTCGTCTGA | GATACAACCACCCTTCCT | 60 | 131 |
| Unigene1509_All | ThERF28 | GATAACGAGACGACAATGG | GACGACTGCTGAAACAAG | 60 | 131 |
| Unigene15444_All | ThERF29 | CGTCATCATCGTCATCATC | GGGTGCTCTAATCTCAGAA | 60 | 107 |
| Unigene16789_All | ThERF30 | GGCAGCACTCAAGTATTG | CTCCTGTCTGGTCATGTT | 60 | 100 |
| Unigene17167_All | ThERF31 | CATAGTCATCAGCCTCAGA | CGTCACACTCGTCAATAAC | 60 | 184 |
| Unigene17341_All | ThERF32 | TTCTCCGTCTTCGTCTTC | CTCCAGCAAGTTGTTATCC | 60 | 130 |
| Unigene17356_All | ThERF33 | TGGAAAGAGTGAGGGAATC | ACCACCACATTCTCTATCC | 60 | 154 |
| Unigene17420_All | ThERF34 | GTCTGGAAGAGTGAATCAAG | AGAAGACGAGTCGGAATC | 60 | 109 |
| Unigene17549_All | ThERF35 | GAATCTGGTTAGGGTCGTA | GGTGCTGAAGGGAAATTG | 60 | 109 |
| Unigene17818_All | ThERF36 | GACACTCTACACCTAAGCA | CCACTTCCTCGTCTTCTT | 60 | 156 |
| Unigene19316_All | ThERF37 | AGAGGAGTTACACGACATAG | GCTGCTTTCTCTTCCTTATC | 60 | 134 |
| Unigene19512_All | ThERF38 | TATTCGTCGTCTCTTCCTG | CTGTCTCACTCCTCTGTATT | 60 | 117 |
| Unigene20038_All | ThERF39 | GCAACAGTCGATGAAGTC | TAGTCCAGTGCTCCAATC | 60 | 120 |
| Unigene20053_All | ThERF40 | TCTGAGTTCCCGAAAGAC | GCAGCCGATGACTTATTC | 60 | 133 |
| Unigene21865_All | ThERF41 | CTGGAGTATGAATGCTGATG | GGAGCACGACTGAATAGA | 60 | 151 |
| Unigene22413_All | ThERF42 | CCTCCTCCTCTTTCTCTTC | TCCTACTCTCATTCCCATTC | 60 | 123 |
| Unigene22539_All | ThERF43 | GACACTCCATCAAGAACAAG | TCTCCTCCAAGTAATCACTC | 60 | 102 |
| Unigene23417_All | ThERF44 | ATCGGAGTATGGCAGAAG | ATCCTGTTGATGAGGTCTC | 60 | 160 |
| Unigene2351_All | ThERF45 | TACTATGTCGGAGGGAGAT | GAGGATGGCACGTTTATTC | 60 | 117 |
| Unigene24800_All | ThERF46 | CAGTCTGAACCAGCATTG | GGCTTCTTCTCCTTCTCTT | 60 | 165 |
| Unigene24813_All | ThERF47 | GAATCATCATCCTTCGGTTC | AATCATCTCGTCGGAGTC | 60 | 105 |
| Unigene24879_All | ThERF48 | TGACAGAGCCGCTTATAG | GTTGACGATGAGGAGGAA | 60 | 135 |
| Unigene25069_All | ThERF49 | GGAGAAGGCGAATAATGC | CTGGTACTGCGTTCATTG | 60 | 144 |
| Unigene25131_All | ThERF50 | GTTTAGAGAGGCGACAATG | GGAGGAGAAGAAGACAGAG | 60 | 115 |
| Unigene25231_All | ThERF51 | GGTGGCTGAGATTAGAGAA | GAAGACGGCGGTATCATA | 60 | 100 |
| Unigene25415_All | ThERF52 | GGCAGCACTCAAGTATTG | CTCCTGTCTGGTCATGTT | 60 | 100 |
| Unigene27354_All | ThERF53 | TCCTCATCACTATGCTCAG | GGTTGTGGAAGAAGAAGAAG | 60 | 142 |
| Unigene27504_All | ThERF54 | CTACTTCTCCGCCGTATT | CGAAACTTCTTCCTCCCA | 60 | 139 |
| Unigene27991_All | ThERF55 | CCTCCTCCTGAAATGTGT | TATGTCGGACTCCTCTGA | 60 | 118 |
| Unigene29462_All | ThERF56 | TATCCTCCAGCGACCTAT | CACAATCCTCCACACTCT | 60 | 171 |
| Unigene29470_All | ThERF57 | TGGCTTATGACAAGGAGG | GTGGTATGGGAGGAATATGA | 60 | 142 |
| Unigene29712_All | ThERF58 | AAGGGAGAGAGCCAATAAG | GGATGCTGAAGGAGATGA | 60 | 157 |
| Unigene29897_All | ThERF59 | GCCTCTGAATGAGAATGAC | GGAGTAGATTGGTGCCTTA | 60 | 108 |
| Unigene300_All | ThERF60 | CATTCTACAGAGGCTACCA | GTGCTATTAGTCCTGCTTTC | 60 | 129 |
| Unigene30691_All | ThERF61 | GGAGTTACCAGGCATAGAT | CAGCTTCCTCGTCATCATA | 60 | 127 |
| Unigene32298_All | ThERF62 | GGATAATACGCTGTTGACTG | GCAGAATCCGATGAATCAC | 60 | 192 |
| Unigene32510_All | ThERF63 | GACTGCTCTACCTCTTCTC | GGAATCAGACACGGATGA | 60 | 144 |
| Unigene32524_All | ThERF64 | GGCAGCACTCAAGTATTG | CTCCTGTCTGGTCATGTT | 60 | 100 |
| Unigene32553_All | ThERF65 | CTTATCCAACGGCTGAGA | CGCCTTCCTAATCTCCAT | 60 | 146 |
| Unigene34083_All | ThERF66 | GGCAGCACTCAAGTATTG | CTCCTGTCTGGTCATGTT | 60 | 100 |
| Unigene3711_All | ThERF67 | TCGTGAAGGAGAAGAACC | CGAATAGCCTCACCATCT | 60 | 171 |
| Unigene755_All | ThERF68 | CTTCTTCTTCACCTCATTCC | GACACCCATTTGTTTCCC | 60 | 136 |
| Unigene774_All | ThERF69 | GGAACAACCACCCACTATA | CGCAACTCTTACCTCTCT | 60 | 179 |
| Unigene958_All | ThERF70 | TACAAGCCTCTCCATTCC | CTTACAATCCTCCGATACCT | 60 | 195 |
| Unigene47493_All | GAPDH | AGCAGCCTTGTCCTTGTCAGTG | GATTGGACGTTTGGTTGCGAG | 60 | 150 |
