# Supplementary material for: Identification of AP2/ERF transcription factors in Tetrastigma hemsleyanum revealed the specific roles of ERF46 under cold stress
Source: Front Plant Sci. 2022 Aug 9;13:936602. doi: 10.3389/fpls.2022.936602 (PMC9396264; doi:10.3389/fpls.2022.936602)
Supplement: Supplementary file 7 [file Table_2.DOCX]

Table S2 All AP2/ERF protein domains analysis.

| Query | Hit type | PSSM-ID | From | To | E-Value | Bitscore | Accession | Short name | Incomplete | Superfamily |
| --- | --- | --- | --- | --- | --- | --- | --- | --- | --- | --- |
| ThERF1 | specific | 197689 | 225 | 276 | 1.16E-15 | 71.1421 | smart00380 | AP2 | - | cl00033 |
| ThERF2 | specific | 197689 | 113 | 173 | 3.91E-21 | 85.3945 | smart00380 | AP2 | - | cl00033 |
| ThERF3 | specific | 197689 | 17 | 78 | 1.6E-34 | 117.366 | smart00380 | AP2 | - | cl00033 |
| ThERF4 | specific | 197689 | 189 | 248 | 1.79E-35 | 124.3 | smart00380 | AP2 | - | cl00033 |
| ThERF5 | specific | 197383 | 171 | 286 | 1.2E-23 | 93.5464 | cd10017 | B3_DNA | - | cl15242 |
| ThERF6 | specific | 237985 | 48 | 105 | 1.06E-18 | 78.8527 | cd00018 | AP2 | - | cl00033 |
| ThERF7 | specific | 197689 | 249 | 312 | 8E-27 | 102.343 | smart00380 | AP2 | - | cl00033 |
| ThERF8 | specific | 237985 | 108 | 149 | 2.75E-24 | 93.4902 | cd00018 | AP2 | C | cl00033 |
| ThERF9 | specific | 197689 | 101 | 159 | 1.93E-31 | 107.351 | smart00380 | AP2 | - | cl00033 |
| ThERF10 | specific | 197689 | 156 | 218 | 5.5E-24 | 93.0984 | smart00380 | AP2 | - | cl00033 |
| ThERF11 | specific | 197689 | 115 | 176 | 2.24E-35 | 120.833 | smart00380 | AP2 | - | cl00033 |
| ThERF12 | specific | 197689 | 7 | 65 | 7.42E-21 | 81.1573 | smart00380 | AP2 | - | cl00033 |
| ThERF13 | specific | 197689 | 75 | 133 | 1.09E-30 | 108.121 | smart00380 | AP2 | - | cl00033 |
| ThERF14 | specific | 402342 | 372 | 480 | 1.24E-62 | 199.013 | pfam10664 | NdhM | - | cl11268 |
| ThERF15 | specific | 197689 | 91 | 149 | 4.15E-33 | 114.284 | smart00380 | AP2 | - | cl00033 |
| ThERF16 | specific | 197689 | 250 | 311 | 5.06E-26 | 100.417 | smart00380 | AP2 | - | cl00033 |
| ThERF17 | specific | 197689 | 329 | 401 | 1.31E-24 | 96.18 | smart00380 | AP2 | - | cl00033 |
| ThERF18 | specific | 197689 | 70 | 127 | 3.63E-33 | 117.751 | smart00380 | AP2 | - | cl00033 |
| ThERF19 | specific | 197689 | 52 | 110 | 3.82E-33 | 115.055 | smart00380 | AP2 | - | cl00033 |
| ThERF20 | specific | 197689 | 74 | 133 | 4.57E-36 | 123.914 | smart00380 | AP2 | - | cl00033 |
| ThERF21 | specific | 197689 | 24 | 85 | 7.05E-29 | 102.343 | smart00380 | AP2 | - | cl00033 |
| ThERF22 | specific | 197689 | 97 | 154 | 2.03E-34 | 121.218 | smart00380 | AP2 | - | cl00033 |
| ThERF23 | specific | 197689 | 111 | 167 | 3.29E-35 | 122.374 | smart00380 | AP2 | - | cl00033 |
| ThERF24 | specific | 197689 | 113 | 169 | 4.31E-34 | 119.677 | smart00380 | AP2 | - | cl00033 |
| ThERF25 | specific | 197689 | 46 | 104 | 2.45E-35 | 119.677 | smart00380 | AP2 | - | cl00033 |
| ThERF26 | specific | 197689 | 111 | 170 | 5.28E-32 | 111.203 | smart00380 | AP2 | - | cl00033 |
| ThERF27 | specific | 197689 | 61 | 117 | 5.58E-31 | 108.506 | smart00380 | AP2 | - | cl00033 |
| ThERF28 | specific | 197689 | 21 | 83 | 1.3E-30 | 106.195 | smart00380 | AP2 | - | cl00033 |
| ThERF29 | specific | 197689 | 33 | 93 | 1.73E-21 | 84.2389 | smart00380 | AP2 | - | cl00033 |
| ThERF30 | specific | 197689 | 329 | 401 | 1.24E-24 | 96.18 | smart00380 | AP2 | - | cl00033 |
| ThERF31 | specific | 197689 | 25 | 88 | 1.17E-20 | 81.1573 | smart00380 | AP2 | - | cl00033 |
| ThERF32 | specific | 197689 | 17 | 76 | 6.54E-20 | 77.6905 | smart00380 | AP2 | - | cl00033 |
| ThERF33 | specific | 197689 | 77 | 141 | 1.02E-21 | 84.2389 | smart00380 | AP2 | - | cl00033 |
| ThERF34 | specific | 197689 | 20 | 82 | 7.82E-23 | 88.0909 | smart00380 | AP2 | - | cl00033 |
| ThERF35 | specific | 197689 | 1 | 57 | 2.3E-23 | 85.7797 | smart00380 | AP2 | - | cl00033 |
| ThERF36 | specific | 197689 | 29 | 85 | 4.63E-21 | 81.1573 | smart00380 | AP2 | - | cl00033 |
| ThERF37 | specific | 197689 | 124 | 188 | 7.87E-26 | 98.106 | smart00380 | AP2 | - | cl00033 |
| ThERF38 | specific | 197689 | 148 | 207 | 6.88E-22 | 86.9353 | smart00380 | AP2 | - | cl00033 |
| ThERF39 | specific | 197689 | 190 | 248 | 1.15E-31 | 113.129 | smart00380 | AP2 | - | cl00033 |
| ThERF40 | specific | 197689 | 122 | 180 | 9.54E-34 | 117.366 | smart00380 | AP2 | - | cl00033 |
| ThERF41 | specific | 197689 | 119 | 182 | 3.46E-33 | 116.981 | smart00380 | AP2 | - | cl00033 |
| ThERF42 | specific | 197689 | 90 | 148 | 1E-32 | 113.899 | smart00380 | AP2 | - | cl00033 |
| ThERF43 | specific | 197689 | 83 | 141 | 8.77E-23 | 86.9353 | smart00380 | AP2 | - | cl00033 |
| ThERF44 | specific | 197689 | 6 | 63 | 5.67E-34 | 115.055 | smart00380 | AP2 | - | cl00033 |
| ThERF45 | specific | 197689 | 17 | 80 | 9.35E-18 | 73.0681 | smart00380 | AP2 | - | cl00033 |
| ThERF46 | specific | 197689 | 24 | 80 | 3.99E-22 | 83.8537 | smart00380 | AP2 | - | cl00033 |
| ThERF47 | specific | 197689 | 116 | 174 | 9.38E-20 | 80.0017 | smart00380 | AP2 | - | cl00033 |
| ThERF48 | specific | 197689 | 138 | 202 | 1.56E-32 | 114.284 | smart00380 | AP2 | - | cl00033 |
| ThERF49 | specific | 197689 | 21 | 78 | 9.64E-20 | 79.6165 | smart00380 | AP2 | - | cl00033 |
| ThERF50 | specific | 197689 | 218 | 280 | 1.05E-34 | 123.914 | smart00380 | AP2 | - | cl00033 |
| ThERF51 | specific | 197689 | 23 | 85 | 7.6E-33 | 110.432 | smart00380 | AP2 | - | cl00033 |
| ThERF52 | specific | 197689 | 329 | 401 | 1.24E-24 | 96.18 | smart00380 | AP2 | - | cl00033 |
| ThERF53 | specific | 197689 | 65 | 123 | 1.32E-35 | 121.603 | smart00380 | AP2 | - | cl00033 |
| ThERF54 | specific | 237985 | 64 | 122 | 5.12E-29 | 103.12 | cd00018 | AP2 | - | cl00033 |
| ThERF55 | specific | 197689 | 76 | 137 | 2.36E-32 | 109.277 | smart00380 | AP2 | - | cl00033 |
| ThERF56 | specific | 197689 | 57 | 115 | 3.54E-33 | 115.055 | smart00380 | AP2 | - | cl00033 |
| ThERF57 | specific | 197689 | 142 | 201 | 2.75E-34 | 120.833 | smart00380 | AP2 | - | cl00033 |
| ThERF58 | specific | 197689 | 106 | 168 | 4.17E-21 | 85.0093 | smart00380 | AP2 | - | cl00033 |
| ThERF59 | specific | 197689 | 61 | 119 | 2.98E-32 | 110.432 | smart00380 | AP2 | - | cl00033 |
| ThERF60 | specific | 197689 | 113 | 177 | 3.25E-31 | 110.047 | smart00380 | AP2 | - | cl00033 |
| ThERF61 | superfamily | 412123 | 70 | 137 | 2.42E-10 | 52.6525 | cl00033 | AP2 superfamily | - | cl00033 |
| ThERF62 | specific | 197689 | 124 | 185 | 1.54E-37 | 126.226 | smart00380 | AP2 | - | cl00033 |
| ThERF63 | specific | 197689 | 99 | 149 | 3.35E-26 | 94.6392 | smart00380 | AP2 | - | cl00033 |
| ThERF64 | specific | 197689 | 432 | 495 | 1.49E-27 | 105.425 | smart00380 | AP2 | - | cl00033 |
| ThERF65 | specific | 237985 | 62 | 120 | 1.24E-28 | 102.735 | cd00018 | AP2 | - | cl00033 |
| ThERF66 | specific | 197689 | 432 | 495 | 1.49E-27 | 105.425 | smart00380 | AP2 | - | cl00033 |
| ThERF67 | specific | 396783 | 193 | 296 | 1.1E-31 | 115.163 | pfam02362 | B3 | - | cl15242 |
| ThERF68 | specific | 197689 | 74 | 129 | 3E-26 | 96.5652 | smart00380 | AP2 | - | cl00033 |
| ThERF69 | specific | 197689 | 74 | 135 | 3.49E-31 | 109.277 | smart00380 | AP2 | - | cl00033 |
| ThERF70 | specific | 197689 | 174 | 233 | 1.23E-35 | 124.3 | smart00380 | AP2 | - | cl00033 |
