# Supplementary material for: Identification of AP2/ERF transcription factors in Tetrastigma hemsleyanum revealed the specific roles of ERF46 under cold stress
Source: Front Plant Sci. 2022 Aug 9;13:936602. doi: 10.3389/fpls.2022.936602 (PMC9396264; doi:10.3389/fpls.2022.936602)
Supplement: Supplementary file 8 [file Table_3.DOCX]

Table. S3 Domain annotations of 15 AP2/ERF TFs in *T. hemsleyanum*.

| Protein ID | Model Similarity | Domain annotation |
| --- | --- | --- |
| ThERF5 | 55.29 | DNA-binding protein RAV1 Solution Structure of the B3 DNA-Binding Domain of RAV1 |
| ThERF14 | 75.18 | NAD(P)H-quinone oxidoreductase subunit M Chloroplast NDH complex |
| ThERF31 | 70.18 | Ethylene-responsive transcription factor 1 Solution NMR Structure of the complex of GCC-box bingding domain of AtERF1 and GCC-box DNA, minimized average structure |
| ThERF32 | 75.86 | Solution structure of the GCC-box binding domain, NMR, 46 structures |
| ThERF35 | 36.99 | AP2/ERF and B3 domain-containing transcription repressor TEM1 Crystal structure of Arabidopsis TEM1 AP2 domain |
| ThERF37 | 50.79 | Ethylene-responsive transcription factor ERF096 Crystal Structure of AtERF96 with GCC-box |
| ThERF40 | 80.33 | Ethylene-responsive transcription factor 1 Solution NMR Structure of the complex of GCC-box bingding domain of AtERF1 and GCC-box DNA, minimized average structure |
| ThERF44 | 65.52 | Ethylene-responsive transcription factor 1 Solution NMR Structure of the complex of GCC-box bingding domain of AtERF1 and GCC-box DNA, minimized average structure |
| ThERF45 | 60.34 | Ethylene-responsive transcription factor 1 |
| ThERF46 | 71.43 | Ethylene-responsive transcription factor ERF096 Crystal Structure of AtERF96 with GCC-box |
| ThERF48 | 98.53 | AtERF1 solution structure of the GCC-box binding domain, NMR, minimized mean structure |
| ThERF49 | 70.18 | Ethylene-responsive transcription factor 1 Solution NMR Structure of the complex of GCC-box bingding domain of AtERF1 and GCC-box DNA, minimized average structure |
| ThERF55 | 68.97 | Ethylene-responsive transcription factor 1 Solution NMR Structure of the complex of GCC-box bingding domain of AtERF1 and GCC-box DNA, minimized average structure |
| ThERF56 | 70.19 | Ethylene-responsive transcription factor 1 Solution NMR Structure of the complex of GCC-box bingding domain of AtERF1 and GCC-box DNA, minimized average structure |
| ThERF67 | 82.41 | DNA-binding protein RAV1 Solution Structure of the B3 DNA-Binding Domain of RAV1 |
